# Linking Patient-Reported and Clinician-Assessed Wound Status via Chatbot-Based Digital Surveillance for Wound Infection: Retrospective Observational Study

**DOI:** 10.2196/77685

**Published:** 2026-01-08

**Authors:** Yung-Cheng Su, Yu-Hsien Lin, Ming-Yuan Huang

**Affiliations:** 1 Emergency Department Chia-Yi Christian Hospital Chia-Yi Taiwan; 2 Department of Plastic Surgery Shin Kong WHS Memorial Hospital Taipei Taiwan; 3 Emergency Department Mackay Memorial Hospital Taipei Taiwan; 4 College of Medicine Mackay Medical University New Taipei City Taiwan

**Keywords:** wound infection, patient-reported symptoms, telemedicine, chatbot, diagnostic accuracy, digital health, artificial intelligence, AI

## Abstract

**Background:**

Digital wound monitoring has become increasingly feasible with the widespread use of smartphones and mobile messaging platforms. Although most previous studies have focused on chronic wounds and demonstrated the clinical benefits of remote monitoring, little is known about how patients with acute wounds perceive and report wound-related changes after discharge; these factors may affect the accuracy and reliability of patient-facing digital health systems.

**Objective:**

This study aimed to evaluate the diagnostic performance of patient-reported infection symptoms in predicting clinician-initiated callbacks within a chatbot-based wound monitoring platform. A secondary objective was to identify wound features most strongly associated with patient-reported infection and examine differences between acute and chronic wound populations.

**Methods:**

This retrospective observational study was conducted at a tertiary medical center in Taipei, Taiwan, between June 30, 2022, and March 1, 2023, as part of an institutional digital health initiative. Within this program, adults with acute or chronic wounds voluntarily joined a chatbot-based monitoring system deployed through the Line messaging app using a bring-your-own-device model. Participants submitted daily symptom reports and wound photographs through the chatbot interface. For each submission, patient self-report of infection served as the primary predictor variable, while an independent review by a senior plastic surgeon determined the reference standard (callback vs no callback). Logistic regression and generalized estimating equation models were applied to account for within-subject correlation, with covariates including age, sex, and wound type. Analyses were performed separately for acute and chronic wounds.

**Results:**

This study included 159 patients; 88 (55.3%) had acute wounds and 71 (44.7%) had chronic wounds. Across the study period, 4764 wound photographs were submitted, with a median of 5 (IQR 2-18) photographs per patient. Diagnostic performance differed by wound type. For acute wounds, the area under the receiver operating characteristic curve was 0.702, with 52.6% sensitivity (95% CI 31.7-72.7) and 87.8% specificity (95% CI 84.7-90.3). For chronic wounds, the area under the receiver operating characteristic curve was 0.907, with 94.9% sensitivity (95% CI 93.3-96.2) and 86.4% specificity (95% CI 85.2-87.5). In symptom correlation analyses, redness was significantly associated with patient-reported infection in the acute wound subgroup (odds ratio [OR] 3.94, 95% CI 1.97-7.90; *P*<.001), whereas in the chronic wound subgroup, both redness (OR 86.35, 95% CI 57.11-130.56; *P*<.001) and skin darkening (OR 358.55, 95% CI 244.79-525.16; *P*<.001) showed significant associations (all *P*<.001).

**Conclusions:**

This study highlights the differences in how patients perceive and report infection-related symptoms between populations with acute and chronic wounds. Lower diagnostic accuracy for acute wounds underscores the influence of limited experience and contextual constraints on patient self-assessment. These findings suggest that patient-facing digital wound monitoring systems should be tailored according to wound chronicity and patient experience, incorporating adaptive feedback and artificial intelligence–assisted screening to enhance patient-reported symptom interpretation.

## Introduction

Digital health interventions have become increasingly feasible with the widespread availability of smartphones and mobile internet connectivity [1-3]. Among these, remote wound monitoring has emerged as a promising approach for postdischarge care, enabling patients to share wound information with clinicians as a personalized, value-added service that enhances continuity of care [4-8] and may help reduce health care costs [9]. Previous studies have shown that home-based telemedicine—encompassing remote telemonitoring and teleconsultation—can improve clinical outcomes, including reductions in rehospitalization [10] and mortality rates [9,11,12]. Contemporary telemedicine tools, such as email, telephone, video calls, and mobile apps, now facilitate the secure transmission of wound photographs, allowing clinicians to perform remote assessments with confidence and demonstrating diagnostic and therapeutic efficacy comparable to conventional face-to-face wound care in terms of healing rate and safety [13].

Beyond photographic information, patient-reported symptoms (PRSs) provide complementary insights into patients’ subjective perceptions of wound changes [14,15]. PRSs and outcome measures are increasingly recognized in wound care as valuable tools that capture patients’ subjective experiences and perceived health status and can support earlier recognition of complications [13,16,17]. Previous studies have demonstrated that PRSs combined with remote wound monitoring can enhance patient-clinician communication and enable more timely intervention, thereby potentially reducing delays in complication detection [5,6,18]. However, most of this work has focused on using PRSs and wound photographs to assist clinician evaluation in remote care. In practice, accurate patient recognition of clinically significant wound changes can reduce unnecessary clinic visits and enable timely intervention [5,7,19], whereas poor accuracy may delay care or generate unnecessary anxiety [20-22].

Building on these previous findings, we developed and implemented a chatbot-based digital wound-monitoring platform that integrates PRS collection with wound photography through the widely used Line messaging app in Taiwan [23]. The system was designed to support continuous postdischarge follow-up and examine how patients perceive wound changes and how these perceptions align with or differ from physicians’ assessments. Understanding this perceptual gap represents a critical step toward advancing patient-centered digital wound care.

Accordingly, this study aimed to (1) evaluate the diagnostic performance of patient-reported infection in predicting physician-initiated callbacks and (2) identify which observable wound characteristics (eg, redness and skin darkening) are most strongly associated with patients’ self-assessments. By characterizing both concordance and discordance between patient and clinician perspectives, this research seeks to inform the design and development of more effective, patient-centered models for digital wound care.

## Methods

### Study Design and Participants

This retrospective observational study was conducted at a tertiary medical center in Taipei, Taiwan, between June 30, 2022, and March 1, 2023. It was nested within a broader institutional digital health initiative launched in 2020 during the COVID-19 pandemic to maintain continuity of care through remote monitoring programs implemented across multiple medical subspecialties. Within the institutional wound care program, adults with acute or chronic wounds were enrolled and followed longitudinally through a chatbot-based digital wound-monitoring platform. Participants voluntarily joined the mobile health program using a bring-your-own-device model and provided complete follow-up data during the study period. Each day, participants submitted symptom reports and wound photographs via the chatbot interface. For each image submission, the patient’s self-reported presence or absence of infection served as the primary predictor variable, while the reference standard was established through an independent review by a senior plastic surgeon with more than 20 years of experience, who classified each case as requiring a callback (follow-up contact) or no callback.

**Figure 1:**
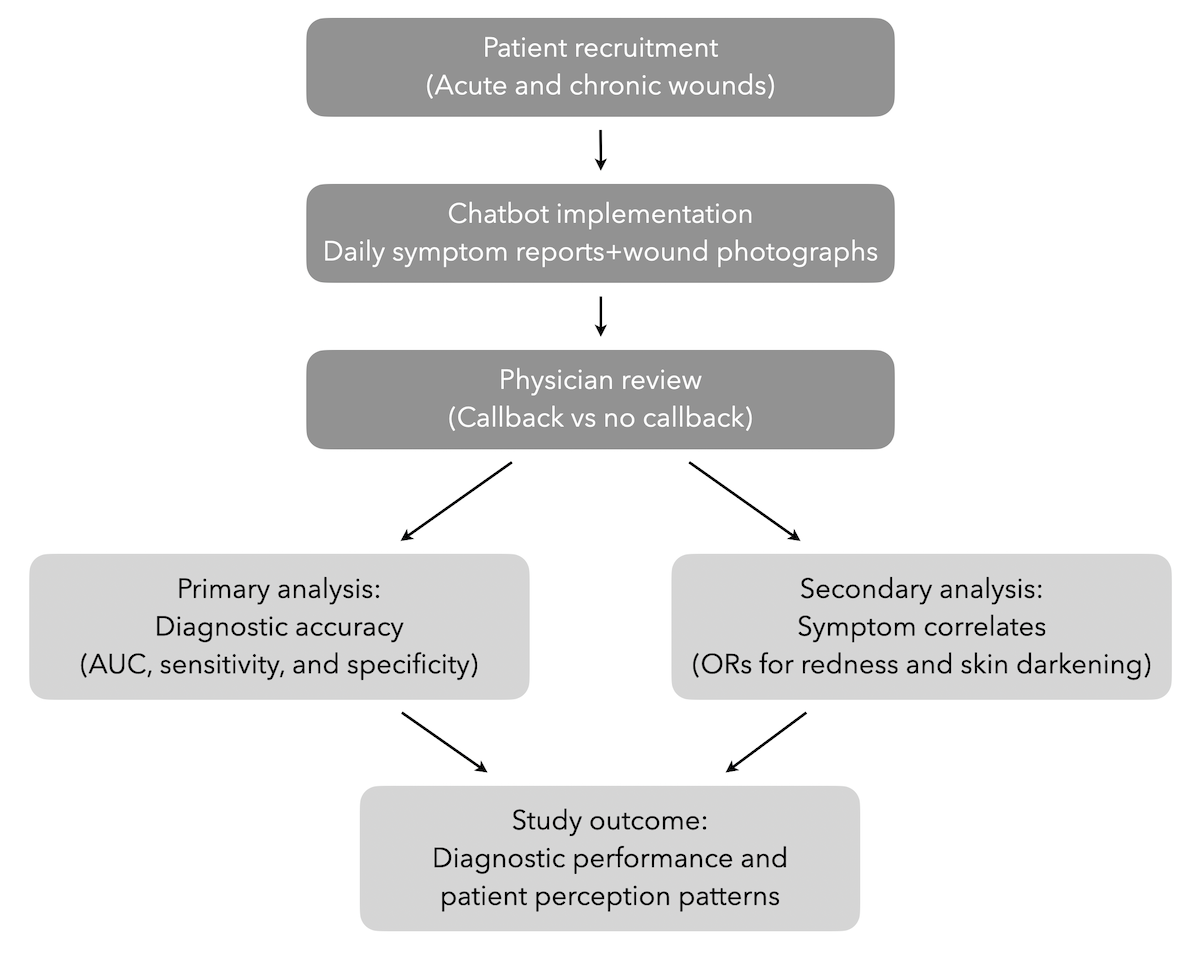
Schematic representation of the study workflow. Patients with acute or chronic wounds were recruited and submitted daily symptom reports and photographs through the chatbot. Physicians subsequently reviewed the data and determined the need for a callback. Primary analyses focused on diagnostic accuracy and secondary analyses examined symptom correlates. AUC: area under the receiver operating characteristic curve; OR: odds ratio.

The overall study workflow is illustrated in Figure 1. The primary analysis evaluated diagnostic accuracy metrics—area under the receiver operating characteristic curve (AUC), sensitivity, and specificity—comparing patient-reported infection with physician-determined callback status. In the secondary analysis, we explored how patients formed subjective judgments of infection by examining coreported symptom descriptors (eg, redness and skin darkening). Because acute and chronic wounds differ inherently in clinical course, symptom dynamics, and expected follow-up duration, they were analyzed as 2 distinct cohorts to account for potential differences in how patients perceive and report infection-related changes. Logistic regression models estimated odds ratios (ORs) for these symptom features associated with the self-reported infection label.

### Patient-Facing Chatbot Design and Implementation

The patient-facing chatbot evaluated in this study was developed as part of the institutional digital health program to support remote wound monitoring and patient self-management. It was designed to enhance postdischarge engagement and facilitate early identification of wound complications through structured symptom reporting and image submission. The development of the chatbot followed a structured, multiphase process grounded in previous literature and expert consensus. First, the chatbot’s architecture and user interactions were developed through an evidence-informed design approach, referencing previous studies on wound care telemonitoring and digital adherence interventions [1,6,24,25]. These studies guided the refinement of message tone, literacy level, and interaction flow to improve comprehension and adherence in remote wound self-monitoring.

Second, an initial prototype was constructed based on the hospital’s existing wound care discharge instructions and supplemented by patient-education materials and discharge guidance documents from other tertiary medical centers in Taiwan. Third, expert consultations were held with a senior plastic surgeon, 2 wound care nurse practitioners, and a digital health usability specialist. These experts reviewed the chatbot’s structure, clinical terminology, and message sequencing through several iterative sessions. Refinements emphasized clarity, consistency with institutional wound care guidance, and user-friendliness for patients with limited digital literacy.

The chatbot interface was implemented through the Line messaging app. Patients accessed the chatbot by scanning a QR code provided at hospital discharge, which led to in-app registration. Once enrolled, participants received 1 automated message per day prompting them to report wound status and upload photographs. Multimedia Appendix 1 provides a representative chatbot-patient conversation flow, illustrating how patients reported wound status and submitted photographs through the Line messaging app interface.

Before full implementation, a 10-patient pilot test was conducted to assess readability, usability, and response compliance [26]. On the basis of feedback, message content was simplified, and the daily symptom reporting options were refined into 3 categories: *normal*, *redness*, and *infection*. This design aimed to balance clinical informativeness with ease of patient interpretation.

Submitted reports and images were automatically stored within a secure cloud database (Amazon Web Services) and reviewed by the wound care team as part of routine remote monitoring. Feedback was provided only when image quality allowed adequate clinical evaluation or when reported symptoms suggested possible deterioration. If wound progression or complications were suspected, the clinical team initiated contact for further evaluation or follow-up scheduling. All chatbot interactions were logged within the broader institutional digital health evaluation program to ensure compliance with data protection and usability standards.

### Data Collection

Patients were recruited from the plastic surgery outpatient clinics and the emergency department at a tertiary care center in Taipei, Taiwan. Eligible patients were introduced to the digital wound monitoring program during their initial clinical visits. Physicians explained the purpose and procedures of the chatbot-based system and invited patients to participate. Enrollment was voluntary and based on convenience sampling. Inclusion criteria were as follows: (1) being aged 18 years or older, (2) having a diagnosis of an acute or chronic wound, and (3) being able to use a smartphone and the Line messaging app. Exclusion criteria were as follows: (1) having an immediate need for surgical revision, (2) declining participation in the digital wound-monitoring program, and (3) having insufficient digital literacy or limited access to a compatible smartphone or stable internet connection.

Once enrolled, participants were followed through a secure mobile chatbot platform that supported asynchronous wound symptom reporting. Patients were instructed to submit daily symptom checklists and wound photographs. Each data entry was time-stamped and linked to a unique patient identifier. The wound care team accessed these inputs via a clinician dashboard, where photo quality was assessed, and clinical decisions regarding follow-up were recorded. All data used in this study were extracted retrospectively from the system database after the study period.

### Statistical Analysis

All data were deidentified before statistical analysis. Age and follow-up duration were treated as continuous variables, while all other covariates were treated as categorical.

For the primary analysis, we evaluated the diagnostic accuracy of patient-reported infection in predicting the need for callback, as determined by the wound care physician. ORs, sensitivity, specificity, and AUC were calculated using physician-determined callback as the reference standard.

For the secondary analysis, we examined the internal consistency of patient-reported infection by evaluating whether these self-reports were systematically associated with coreported symptom descriptors (eg, redness and skin darkening) across repeated submissions. Each photo level PRS entry was dichotomized as *infection* or *no infection*, and regression models were used to estimate the strength of these associations. Consistent relations across time would indicate that patients applied a stable internal criterion when identifying infection, whereas the absence of such patterns would suggest inconsistency in self-assessment. We used generalized estimating equations (GEEs) to account for the clustering of multiple photograph submissions per patient, which would otherwise violate the independence assumption of standard regression models. This approach accounted for the within-subject correlation in repeated submissions and yielded variance-adjusted SE estimates and approximately valid CIs under the assumed correlation structure. Given the convenience sampling design, available demographic and clinical covariates—age, sex, and wound type (acute vs chronic)—were included in all models to mitigate potential confounding. GEE models were used in both primary and secondary analyses. However, other potentially influential factors, such as comorbidity burden, wound etiology, and patients’ digital literacy, were not systematically collected in the retrospective dataset and could therefore not be adjusted for.

All analyses were conducted using SAS (version 9.4; SAS Institute Inc) for Windows. A 2-tailed *P* value of <.05 was considered statistically significant.

### Ethical Considerations

This study was approved by the institutional review board of the Shin Kong WHS Memorial Hospital, Taipei, Taiwan (approval 20211001R). The requirement for written informed consent was waived because of the retrospective study design involving previously collected clinical photographs. All data were anonymized before analysis, and confidentiality was maintained throughout the study. All images and multimedia materials included in the manuscript have been carefully reviewed and deidentified before publication. No financial or material compensation was provided to participants.

## Results

### Overview

A total of 159 patients were included in the final analysis, of whom 88 (55.3%) were classified as acute wound cases. On average, the patients were aged 45.6 (SD 1.25) years. Throughout the study period, 4764 wound photographs were submitted through the mobile digital health platform, with a median of 5 (IQR 2-18) photographs per patient. Among these, patients reported suspected infection in 1352 (28.4%) instances, redness in 1542 (32.4%) instances, and skin darkening in 1040 (21.8%) instances. The detailed demographics are summarized in Table 1.

**Table 1:** Baseline characteristics of patients and wound photographs.^a^

Patient characteristics	Overall (N=159)	Acute wound (n=88)	Chronic wound (n=71)
Age (y), mean (SD)	45.6 (1.25)	35.5 (1.14)	58.0 (2.21)
Sex: male, n (%)	94 (59.1)	56 (63.6)	38 (53.5)
Total photos uploaded, n	4764	559	4205
Uploaded photos per patient, mean (SD)	30.0 (7.16)	6.4 (0.89)	59.2 (15.48)
Patient reported suspected infection, n (%)	1352 (28.4)^b^	76 (13.6)^c^	1276 (30.3)^d^
Redness, n (%)	1542 (32.4)^b^	252 (45.1)^c^	1290 (30.7)^d^
Skin darkening, n (%)	1040 (21.8)^b^	129 (23.1)^c^	911 (21.7)^d^
Callbacks, n (%)	886 (18.6)^b^	19 (3.4)^c^	867 (20.6)^d^

^a^Values are presented as mean (standard deviation, SD) for continuous variables and n (%) for categorical or photograph-level variables.

^b^n=4764.

^c^n=559.

^d^n=4205.

### Primary Analysis: Diagnostic Performance of PRS-Reported Infection for Predicting Callbacks

A total of 1352 wound images were marked by patients as showing infection in the symptom checklist. Among these, 886 (65.5%) images were also classified as requiring callback by the reference physician reviewer and were used to evaluate diagnostic performance. Overall, patient-reported infection demonstrated strong diagnostic accuracy for predicting physician-initiated callbacks, with an overall sensitivity of 94% (95% CI 92.3-95.4) across all cases.

**Figure 2:**
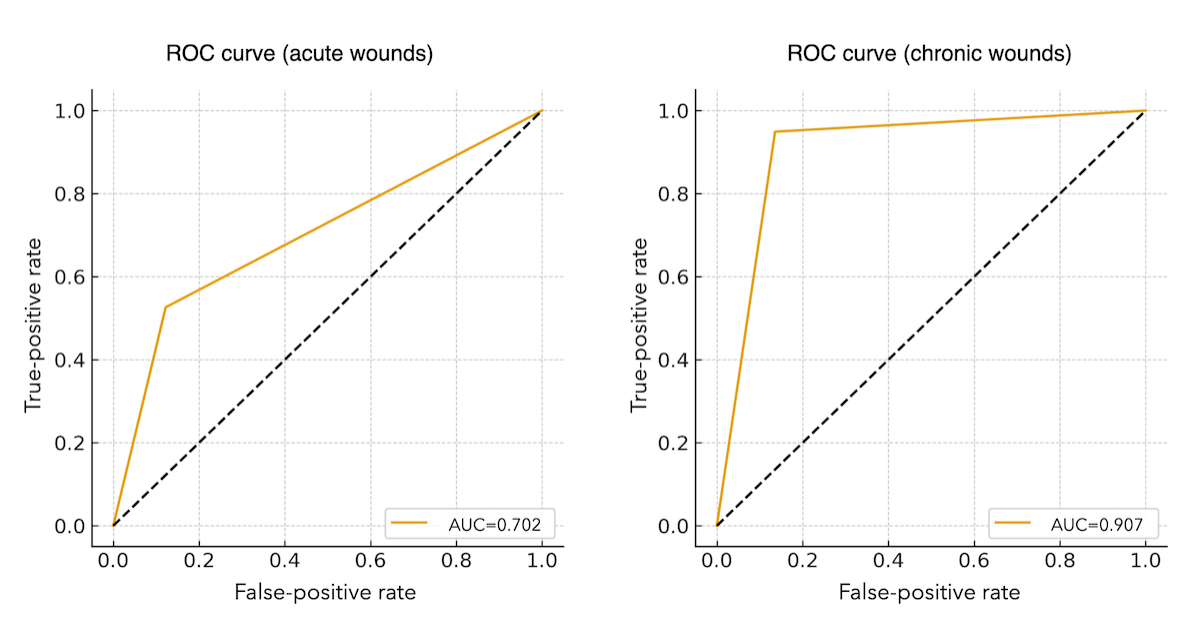
Diagnostic accuracy of patient-reported infection for predicting physician-initiated callbacks among acute and chronic wound patients. Receiver operating characteristic (ROC) analysis shows reduced discriminative performance, with an area under the curve (AUC) of 0.702 for acute wounds and 0.907 for chronic wounds.

When stratified by wound type, diagnostic performance differed markedly between the 2 cohorts (Figure 2). For acute wounds, the AUC was 0.702, corresponding to a sensitivity of 52.6% (95% CI 31.7-72.7) and a specificity of 87.8% (95% CI 84.7-90.3). This indicated only modest accuracy of patient self-assessment in acute wound contexts. The positive predictive value (precision) for the acute wound group was comparatively low, indicating a higher proportion of false-positive results. The wide CIs observed for this subgroup further reflected its small sample size and low callback rate; therefore, the results should be interpreted with caution. In contrast, chronic wounds demonstrated substantially higher diagnostic performance, with an AUC of 0.907, sensitivity of 94.9% (95% CI 93.3-96.2), and specificity of 86.4% (95% CI 85.2-87.5). The receiver operating characteristic curve for chronic wounds exhibited a steeper initial rise, reflecting a higher true-positive rate at lower false-positive thresholds and greater alignment between patient-assessed and physician-assessed infections.

To account for repeated photo submissions per patient, we fitted a multivariable GEE model as an exploratory analysis (Table 2). After adjusting for age, sex, and wound type (acute vs. chronic), PRS-reported infection remained a statistically significant predictor of callback (adjusted OR 113.65, 95% CI 24.44-528.48; *P*<.001). However, the wide CIs reflected the limited sample size and potential instability of the estimates. For comparison, a conventional logistic regression model without GEE adjustment yielded narrower CIs but may underestimate variance by ignoring intrapatient correlation. The non-GEE results are provided in Table S1 in Multimedia Appendix 2.

**Table 2:** Multivariable generalized estimating equation estimates for predicting physician-initiated callback.

Variable	Adjusted odds ratio (95% CI)	*P* value
Patient-reported symptom–reported infection	113.65 (24.44-528.48)	<.001
Age (y)	1.06 (1.03-1.09)	<.001
Sex: male	0.99 (0.20-5.04)	.99
Wound type: acute	0.26 (0.07-0.98)	.047

### Secondary Analysis: Symptom Correlates of Patient-Reported Infection

To examine the internal consistency of patient-reported infection assessments, we analyzed whether the selection of infection was associated with other simultaneously reported symptom features in each wound subgroup (Table 3). In the acute wound cohort, redness was significantly associated with patient-reported infection (OR 3.94, 95% CI 1.97-7.90; *P*<.001), indicating that patients who noted redness were nearly 4 times more likely to label their wounds as infected. Other features, including skin darkening (OR 0.42, 95% CI 0.10-1.79), age (OR 1.01, 95% CI 0.99-1.04), and sex (OR 0.73, 95% CI 0.33-1.59), were not significantly associated with infection reporting.

In contrast, in the chronic wound cohort, both redness (OR 86.35, 95% CI 57.11-130.56; *P*<.001) and skin darkening (OR 358.55, 95% CI 244.79-525.16; *P*<.001) were strongly associated with infection reports (all *P*<.001), whereas age (OR 1.03, 95% CI 1.02-1.03; *P*=.01) and male sex (OR 1.53, 95% CI 1.10-2.13; *P*=.01) also showed modest but significant associations (all *P*=.01). These findings suggested that patients with chronic wounds demonstrated more consistent symptom-infection linkages, particularly between wound color changes and perceived infection.

**Table 3:** Odds ratios (ORs) for symptom correlates of patient-reported infection.

Effect	Acute wound, OR (95% CI)	Chronic wound, OR (95% CI)
Redness	3.94 (1.97-7.90)	86.346 (57.11-130.56)
Skin darkening	0.42 (0.10-1.79)	358.548 (244.79-525.16)
Age	1.01 (0.99-1.04)	1.025 (1.02-1.03)
Sex: male	0.73 (0.33-1.59)	1.534 (1.10-2.13)

## Discussion

### Overview

This study evaluated a chatbot-facilitated PRS system for remote wound monitoring and identified distinct diagnostic performance patterns between populations with acute and chronic wounds. The subsequent sections discuss these subgroup differences, underlying mechanisms of diagnostic discordance, and their implications for digital integration in real-world wound care.

### Early Identification Through Patient Self-Assessment

Patient-reported infection demonstrated meaningful clinical utility for supporting remote triage; however, its diagnostic performance varied notably between wound types. In the chronic wound cohort, sensitivity was high, and patient assessments were largely concordant with clinician callback decisions, whereas in the acute wound cohort, sensitivity was lower (52.6%) and agreement with clinician judgment was less consistent. These differences underscore that the reliability of patient-reported assessments is strongly influenced by wound chronicity and patient familiarity with symptom trajectories.

In the acute wound subgroup, infection reporting was primarily driven by overt visual cues, such as redness. This suggests that patients with acute wounds can recognize obvious inflammatory changes but may overlook or misinterpret subtler alterations, leading to variability in self-assessment. Their limited experience and lack of reference points—typical of postsurgical or trauma contexts—may constrain their ability to differentiate normal healing responses from early infection [21,27]. The lower precision and wider CIs observed in this subgroup are also likely attributable to the smaller sample size and lower callback event rate, which limit the statistical stability of performance estimates. Therefore, findings from the acute wound subgroup should be interpreted with caution, and future studies with larger cohorts are needed to validate these preliminary observations.

By contrast, the chronic wound cohort demonstrated stronger alignment between patient-reported infection and clinician callback decisions. Patients with chronic wounds appeared to recognize a broader spectrum of wound color changes as potential infection indicators, suggesting the development of experiential knowledge through long-term wound observation [14,20,27]. Taken together, these subgroup differences underscore that PRS-based surveillance tools should be context aware and dynamically adapted to different user populations and clinical environments.

### Discrepancies and Possible Explanations for Diagnostic Discordance

Diagnostic discrepancies between patient-assessed and clinician-assessed infection were evident across wound types and provided insight into the behavioral mechanisms underlying differences in diagnostic precision. In the acute wound cohort, patient self-assessment showed only modest sensitivity and low precision (positive predictive value), reflecting a tendency toward false-positive reporting. Patients in this group often interpreted normal postoperative inflammation—such as transient redness or mild swelling—as signs of infection, likely due to limited experience and heightened vigilance immediately after injury or surgery [21,27,28]. These perceptual and emotional factors may help explain why patients with acute wounds frequently overreported infection despite the absence of clinician-confirmed pathology [18,20].

By contrast, the chronic wound cohort demonstrated high sensitivity and more balanced specificity, indicating strong alignment between patient and clinician assessments. Patients with chronic wounds appeared to interpret visual cues, such as redness and discoloration, in a more calibrated manner, likely reflecting experiential learning from long-term self-observation and repeated feedback [20,27]. This familiarity may enable them to better distinguish stable wound conditions from early signs of deterioration.

Beyond these behavioral differences, several factors may further contribute to the observed diagnostic discordance. First, limited symptom literacy could represent a key underlying contributor; patients without medical knowledge may misinterpret normal inflammatory changes or fail to recognize early indicators of infection, a challenge commonly noted in previous self-monitoring research [4,21,27]. Second, patients often assess wounds predominantly through visual inspection, without access to tactile or contextual cues that typically inform clinical reasoning [25,28]. Third, home-based assessments are likely influenced by practical constraints such as poor lighting, rushed circumstances, or inconsistent photographic quality—which may introduce additional variability [18,22,25]. Finally, previous illness experience and emotional state appear to influence how patients interpret wound changes; individuals without a history of complications tend to underreport symptoms, whereas those who are anxious about infection may overreport, potentially contributing to both false-negative and false-positive results [3,20,27].

Collectively, these findings extend previous research on digital wound monitoring, which has largely focused on chronic wound populations [13,29] and has paid limited attention to how perceptual or interpretive processes may differ across care contexts [22,28]. Our results suggest that such differences become particularly salient in acute wound settings, where patients encounter distinct cognitive and contextual challenges when interpreting wound changes. These findings underscore the importance of contextualizing PRS systems according to wound chronicity and patients’ experience.

### Clinical Implications and Future Directions

This study supports the integration of chatbot-facilitated PRS systems into routine wound care as a scalable strategy for extending surveillance beyond traditional clinical settings. The high sensitivity observed suggests strong potential clinical utility as a triage tool, particularly for patients with chronic wounds requiring ongoing monitoring. However, the lower accuracy observed in acute wounds highlights the need for tailored approaches—such as real-time image quality checks [25] or artificial intelligence (AI)–assisted design [18]—to improve performance in contexts where subjective interpretation may be less reliable.

Although some may have questioned the necessity of PRSs when image-based AI tools are available, the 2 modalities serve complementary rather than redundant roles. PRSs capture subjective and longitudinal information—such as pain, odor, drainage, and daily symptom changes—that images alone cannot provide. When combined with automated image analysis, PRSs can enhance contextual understanding, flag discrepancies between visual and symptomatic cues, and facilitate earlier patient engagement. Because chatbot-based PRS collection incurs minimal marginal cost once deployed [1,9], the potential to prevent infection-related revisits may offset additional resource requirements [8,19]. Future studies should formally evaluate the economic impact and optimal integration of PRSs with image-based AI systems.

### Limitations

Several limitations must be acknowledged. First, the use of convenience sampling may limit generalizability, as participants with greater digital literacy were more likely to enroll. Second, the statistical approach also warrants caution. Given the small sample size and correlated structure of repeated submissions, the GEE model should be interpreted cautiously [30]. Although a non-GEE logistic regression yielded more stable CIs, it could not account for within-patient clustering. Therefore, we retained the GEE model as the primary analysis but emphasized its exploratory nature when interpreting the results.

Third, all callback decisions were made by a single senior plastic surgeon reviewing the chatbot dashboard. Although this ensured consistency, it may also introduce interpretive bias and limit the generalizability of our comparator standard. Additionally, reliance on patient-captured photographs introduces variability in image quality that could affect both patient and clinician assessments [25,28]. Fourth, participants received standard printed wound care education materials outlining routine care procedures and warning signs but did not receive any instruction that defined or exemplified redness or skin darkening. No photographic exemplars or audiovisual materials were provided, which may have influenced secondary analyses that depend on patient interpretation of wound color changes. This lack of targeted symptom education may have affected how patients interpreted visual changes and contributed to variability in self-reported infection assessments [20]. Future work should evaluate whether integrating structured visual education—through images, short videos, or in-app microlearning modules—can improve the consistency and accuracy of patient-reported data.

Fifth, we were unable to adjust for additional potential confounders, such as health literacy, app use patterns, comorbidities, wound severity, or physician workload, as these were not systematically collected. We also did not systematically measure participants’ digital literacy, internet access, or previous experience with social messaging apps, which may have influenced engagement. Observations during recruitment suggest these factors could play a role, but this hypothesis requires formal evaluation in future studies. Finally, subgroup analyses, such as the acute wound cohort, were based on fewer photographs per patient, which may explain the lower AUC observed in this subgroup relative to the overall sample. Moreover, although this study evaluated the diagnostic alignment between patient reports and clinician decisions, it did not assess patient-centered outcomes, such as healing time, complication rates, or patient satisfaction. These outcomes should be addressed in future longitudinal studies. Incorporating qualitative feedback and equity-focused evaluations will also be essential for optimizing system design and ensuring broader applicability.

### Conclusions

This study demonstrates the feasibility and clinical value of a chatbot-facilitated PRS system for remote wound monitoring. PRS-based assessments, when contextualized by wound type, can meaningfully reflect clinical conditions and support early identification of cases requiring follow-up. Although patients with chronic wounds showed high diagnostic concordance with clinician evaluations, patients with acute wounds displayed greater variability, underscoring the importance of tailoring digital surveillance tools to different care contexts. These findings highlight the complementary role of PRSs alongside image-based analysis in enhancing diagnostic consistency and patient engagement. Future work should focus on integrating adaptive educational modules and hybrid PRS-AI models to improve accuracy, efficiency, and scalability in real-world wound care.

## Appendix

Multimedia Appendix 1 Video walkthrough of patient-reported symptom entry via chatbot.

Multimedia Appendix 2 Comparison of logistic regression results without generalized estimating equation adjustment for repeated observations.

## Abbreviations

AI: artificial intelligence
AUC: area under the receiver operating characteristic curve
GEE: generalized estimating equation
OR: odds ratio
PRS: patient-reported symptom

## Data Availability

The datasets generated and/or analyzed during this study are not publicly available due to patient privacy and institutional restrictions but are available from the corresponding author on reasonable request. The full image dataset cannot be made publicly available to protect participant privacy. Deidentified data underlying the study findings may be made available from the corresponding author on reasonable request and with appropriate institutional and ethical approval.
