# Supplementary material for: Linking Patient-Reported and Clinician-Assessed Wound Status via Chatbot-Based Digital Surveillance for Wound Infection: Retrospective Observational Study
Source: JMIR Form Res. 2026 Jan 8;10:e77685. doi: 10.2196/77685 (PMC12828308; doi:10.2196/77685)
Supplement: Multimedia Appendix 2 [file formative_v10i1e77685_app2.docx]

# Supplementary Table 1. Comparison of logistic regression results without GEE adjustment for repeated observations.

|  | OR | 95% CI | p-value |
| --- | --- | --- | --- |
| PRS reported infection | 186.65 | 133.59 - 260.76 | <0.001 |
| Age | 1.06 | 1.05 - 1.07 | <0.001 |
| Gender (male) | 1.48 | 1.15 - 1.90 | 0.024 |
| Wound type (acute) | 0.32 | 0.18 - 0.55 | <0.001 |
